# Single-stage treatment of chronic localized tibial osteomyelitis with local debridement and antibiotic-loaded calcium sulfate implantation: a retrospective study of 42 patients

**DOI:** 10.1186/s13018-020-01721-7

**Published:** 2020-06-01

**Authors:** Chun-Hao Zhou, Ying Ren, Abdulnassir Ali, Xiang-Qing Meng, Hong-An Zhang, Jia Fang, Cheng-He Qin

**Affiliations:** 1 Department of Orthopaedics and Traumatology, Provincial Key Laboratory of Bone and Cartilage Regenerative Medicine, Nanfang Hospital, Southern Medical University, Guangzhou, 510515 People’s Republic of China; 2 Department of Nursing, Nanfang Hospital, Southern Medical University, Guangzhou, 510515 People’s Republic of China; 3 Department of Orthopaedics and Traumatology, Guangdong Second Provincial General Hospital, Guangzhou, 510317 People’s Republic of China

## Abstract

**Background:**

Although various methods have been introduced, the management of chronic tibial osteomyelitis remains a challenge. This study aims to assess a combined treatment method, local debridement combined with antibiotic-loaded calcium sulfate implantation, for the management of the local (Cierny-Mader type III) tibial osteomyelitis.

**Methods:**

Forty-two patients (43 limbs) with type III tibial osteomyelitis, from January 2012 to December 2018, who received the treatment method mentioned above were included in the study. The infection remission rate, recurrence rate, complications rate, and bone healing rate were respectively analyzed.

**Results:**

With a mean follow-up of 42.8 months, 38 limbs (37 patients) (88.4%, 38/43) achieved infection remission without recurrence. Among those patients pain, limitation of movement, sinus tracts, topical redness, and swelling were generally eliminated. Only 4 patients felt slight pain after a long-distance walk, while another 6 patients showed minor but acceptable discomfort in affected limbs. Five patients (11.6%) suffered from osteomyelitis recurrence that required secondary surgical and medical treatment, but no amputation was necessary to eliminate the infection. Prolonged aseptic drainage was the most frequent complication that was observed in 13 patients (30.0%). They were successfully managed by appropriate wound caring in 10 patients and by surgical intervention, months later, in 3 patients. According to the final X-ray examination, bone losses caused by local debridement were generally repaired, though the shape of the tibia was not well-restored to its initial form in 17 limbs. No fracture was recorded during follow-up.

**Conclusion:**

Local debridement combined with antibiotic-loaded calcium sulfate implantation is effective and safe in a single-stage treatment of chronic Cierny-Mader III tibial osteomyelitis.

## Background

Chronic tibial osteomyelitis is defined as a long-term infection of the tibia and characterized by low-grade inflammation with sequestrum or fistulous tract [1]. Secondary to the increased trauma, inappropriate application of implants, or prolonged hematogenous infection, it has become a less rare disease the orthopedists have to face. Once established chronic tibial osteomyelitis, patients are prone to suffere from a variety of disastrous complications, such as pathological fracture, delayed healing or nonunion, or even major amputation, significantly reducing individual’s quality of life. Therefore, immediate and appropriate management is vital for patients with chronic tibial osteomyelitis. Unfortunately, even standard treatment protocols have been strictly carried out, chronic tibial osteomyelitis remains a refractory disease with a noticeable recurrent rate of 20 to 30% [2].

In order to help planning the surgical strategy and improving the effects of treatment, a physiology- and anatomy-based osteomyelitis classification system was introduced by Cierny and Mader in 1985 [3], which now has been widely accepted as the standard classification for chronic long bone osteomyelitis. According to anatomic osseous involvement [4], Cierny et al. primarily divided chronic osteomyelitis into four types: medullary (type I), superficial (type II), localized (type III), and diffused (type IV), and further classified patients into three groups: healthy patients (group A), compromised patients (group B), and patients who were too weak to receive surgery (group C), based on the physiologic status of patients. Attributing to the limited involvement, localized tibial (Cierny-Mader (C-M) type III) osteomyelitis is not as complicated as the diffused one (type IV), but still owning its characteristics. Since the extent of infection is comparatively limited, the clinical symptoms and presentations might not be as severe as the diffused osteomyelitis, which means a better outcome could be achieved if treated immediately and appropriately. In the aspect of surgical management, local debridement without segmental bone resection as the standard surgical management allows the restoration of a healthy enough bone, which avoids the massive bone loss and tedious secondary reconstruction to restore the length of the tibia [5, 6].

With regard to the dead space, second-stage muscle flaps or autogenous cancellous bone grafts after weeks of systemic antibiotics administration are traditional but effective treatment methods [6, 7]. However, their drawbacks lie in the necessity of multiple staged and complicated surgical management, long-term systemic antibiotics application, and potential risk of severe donor sites complications, all of which inevitably increase burdens on both doctors and patients. To overcome those drawbacks, topical antibiotics carrier is a promising method. Highly purified calcium sulfate as the most common biodegradable antibiotic-impregnated material is widely used in the treatment of osteomyelitis in recent decades, and the overall results were good [8, 9]. After implantation, it is associated with the advantages of more accurate positioning, higher local concentration, less side effects and longer treatment duration as well as its potential osteoconductive property [10, 11], while overcoming the shortcomings of non-biodegradable antibiotic carriers.

Statistically, the tibia is the most common site for chronic osteomyelitis to occur [7, 12], partially because of its poor blood supply (especially inferior third of tibia), inadequate coverage in the medial surface, higher risk of injuries, and of course, the inappropriate surgical managements. C-M Type III chronic tibial osteomyelitis as a less severe type has not yet received much attention as C-M type IV and thus, is not frequently illustrated by a single study. Separate studies on localized tibial osteomyelitis alone are still not substantiated. Our study is designed to assess the outcomes of local debridement combined with a substitute of autologous bone, the antibiotic-loaded calcium sulfate, as a single-stage treatment of localized chronic tibial osteomyelitis.

## Patients and methods

### Patients and preoperative management

From January 2012 to December 2018, hundreds of patients with chronic tibia osteomyelitis were treated in our center, but only those meeting the following criteria were included in this study: (1) diagnosed with C-M type III chronic tibial osteomyelitis and treated in our department, (2) completed follow-up of a minimum of 12 months, and (3) the surgery performed was fenestration and debridement, combined with the placement of antibiotic-loaded calcium sulfate. On the contrary, patients with host-C class or received other treatment methods were excluded from the study.

Totally, 42 patients with 43 limbs [24 men and 18 women (19 limbs)], with an average age of 43.7 years (range, 23–74 years), met the criteria and were included for analysis. The median duration of osteomyelitis was 6 months (range, 2–300 months). There were 24 (55.8%) focal infections on left tibias, and 19 (44.2%) on the right tibias. At least 2 patients were recorded as a smoker, and the other 2 patients were diagnosed with hypertension. Regarding the physiologic status of the patients, 35 patients (36 limbs) were defined as C-M type IIIA compared to 7 patients as C-M type IIIB (6 cases with type IIIB^S^ and 1 with type IIIB^L^). While the majority of the cases were accompanied with pain, draining sinus, swelling, and slight movement limitation, 14 patients presented with topical pain, redness, or swelling only. The osteomyelitis diagnosis of those cases was initially suspected by recurrent pain and/or rise in temperature at topical sites, and eventually diagnosed by combining topical symptoms, positive imaging examinations, and raised inflammation markers. During the patients’ first physical examination, we did not find any severe soft tissue defects on involved limbs. According to patients’ medical history, trauma and inappropriate treatment several months or years ago was the number one cause for infection (31 limbs), which could further be divided into open tibial fracture (12 limbs, in which 11 limbs received debridement and internal fixations) and closed fracture with ORIF (19 limbs). Hematogenous infection as the second common cause of infection was generally recorded in 10 limbs. Followed by continuous penetration from soft tissue infection in third place (2 limbs). The details of all patients are presented in Table 1.

**Table 1:** Preoperative characteristics and follow-up outcomes of 42 patients (43 limbs)

No.	Age/sex	Side	Infected by	Cigarette/alcohol abuse	Systemic disease	Positive inflammatory markers	C-M classification	Organisms	Flap	External fixation	Hospital stay (days)	Follow-up (months)	Outcomes
1	46/F	L	Trauma	-	-	ESR	III A	-	-	-	6	35.9	Remission, slight pain
2	44/M	L	Trauma	-	-	-	III A	*S*. *aureus*	-	-	9	34.3	Remission
3	24/M	R	Trauma	-	-	-	IIIA	-	-	-	7	32.7	Remission
4	63/M	R	Trauma	-	-	-	III A	-	-	-	8	33.1	Remission
5	40/F	L	Trauma	-	-	ESR	IIIA	-	-	-	9	32.9	Remission, joint stiffness
6	32/F	L	Trauma	-	-	-	IIIA	-	-	-	10	30.9	Remission
7	45/M	L	Trauma	-	-	-	IIIA	*S*. *aureus*	-	Yes	13	26.4	Remission
8	58/M	L	Hematogenous	Cigarette	-	WBC, ESR	III B^S^	*S*. *aureus*	-	-	13	24.9	Remission
9	25/F	L	Hematogenous	-	-	ESR	III A	-	-	-	15	36.4	Remission, fibrous scar formation
10	40/F	R	Hematogenous	-	-	ESR	III A	-	-		11	32.7	Remission
11	43/M	R	Trauma	-	-	-	IIIA	-	-	-	10	40.9	Redebridment and bone transport
12	31/F	R	Hematogenous	-	-	-	III A	-	-	-	17	24.1	Remission, skin traction to cover wound
13	60/F	L	Trauma	-	-	-	III A	-	-	-	15	38.2	Remission
14	61/M	L	Trauma	-	-	ESR	III A	*P*. *Aeruginosa*, *E*. *faecalis*	Yes	Yes	37	89.9	Redebridment and bone transport
15	16/F	R	Trauma	-	-	-	III A	*S*. *aureus*	-	-	10	70.8	Remission
16	74/F	L	Trauma	-	-	ESR	III B^S^	*S*. *aureus*	-	Yes	20	75.8	Remission
17	24/F	R	Trauma	-	-	ESR	III B^S^	-	-	Yes	32	73.2	Redebridment and bone transport
18	53/M	L	Trauma	-	-	ESR	III A	*A*. *baumannii*	-	Yes	46	77.5	Remission
19	26/M	R	Trauma	-	-	-	III A	*S*. *aureus*	-	-	8	67.2	Remission
20	57/F	R	Trauma	-	Hypertension	-	III A	*K*. *pneunoniae*	-	-	12	49.9	Remission
21	46/M	R	Hematogenous	-	-	ESR	III A	-	-	-	11	48.5	Remission
22	59/F	L	Penetration	-	Hypertension	ESR	IIIB^L^	*E*. *cloacae*	-	-	8	19.6	Redebridment and flap coverage
23	23/M	R	Trauma	-	-	-	III A	*S*. *aureus*	-	-	7	68.5	Remission
24	25/M	L	Hematogenous	-	-	-	III A	*E*. *faecalis*	-	-	13	59.6	Remission
25	46/M	L	Trauma	-	Diabetes	ESR	III B^S^	*P*. aeruginosa	-	Yes	29	52.7	Remission
26	44/M	L	Trauma	-	-	-	III A	-	-	Yes	33	47.6	Redebridment and bone transport
27	42 M	L	Trauma	-	-	-	III A	*S*. *aureus*	-	-	9	37.3	Remission
28	30/M	R	Trauma	Cigarette	-	-	III B^S^	-	-	Yes	12	46.4	Remission
29	54/M	R	Trauma	-	-	-	III A	S. *haemolyticu*s	-	Yes	17	43.4	Remission
30	39/F	R	Hematogenous	-	-	-	III A	-	-	-	7	39.3	Remission
31	40/F	L	Hematogenous	-	-	-	III A	-	-	-	11	28	Remission
32	26/M	L	Hematogenous	-	-	ESR	III A	-	-	Yes	16	42.1	Remission
33	63/F	L	Hematogenous	-	-	ESR	III A	*S*. *aureus*	-	-	23	70.7	Remission, slight pain
34	39/F	L	Penetration	-	-	ESR	III A	-	-	-	8	53.6	Remission, slight pain
35	64/M	R	Penetration	-	-	ESR	IIIB^S^	*E*. *coli*	Yes	-	14	72.5	Remission, Powerlessness
36	30/M	R	Trauma	-	-	-	III A	*S*. *aureus*	-	-	11	44.8	Remission, discomfort
37	48/M	R	Trauma	-	-	ESR	III A	*S*. *aureus*	-	-	24	50.9	Remission
38	41/F	R	Trauma	-	-	ESR	III A	-	-	Yes	32	12.8	Remission, claudication, discomfort
39	64/M	L	Trauma	-	-	ESR	III A	*A*. *hydrophila*	-	Yes	22	12.9	Remission
40	61/M	L	Trauma	-	-	ESR	III A	-	-	-	18	14.2	Remission
41	43/F	L	Hematogenous	-	-	-	III A	-	-	-	16	15.6	Remission, slight pain
42	27/F	R	Trauma	-	-	ESR	III A	*P*. *aeruginosa*	-	-	22	16.3	Remission, Slight Discomfort
43	64/F	L	Trauma	-	-	ESR	III A	-	-	Yes	22	17.5	Remission

*Abbreviations*: *M* Male, *F* Female, *L* Left, *R* Right, *WBC* white blood cell, *ESR* erythrocyte sedimentation rate, *CM* calcium sulfate; *S*. *aureus*, *Staphylococcus aureus*; *S*. *haemolyticus*, *Staphylococcus haemolyticus*; *P*. *aeruginosa*, *Pseudomonas aeruginosa*; *K*. *pneunoniae*, *Klebsiella pneunoniae*; *E*. *faecalis*, *Enterococcus faecalis*; *E*. *coli*, *Escherichia coli*; *A*. *hydrophila*, *Aeromonas hydrophila*, *E*. *cloacae*, *Enterobacter cloacae*; *S*. *marcescens*, *Serratia marcescens*

**Fig. 1:**
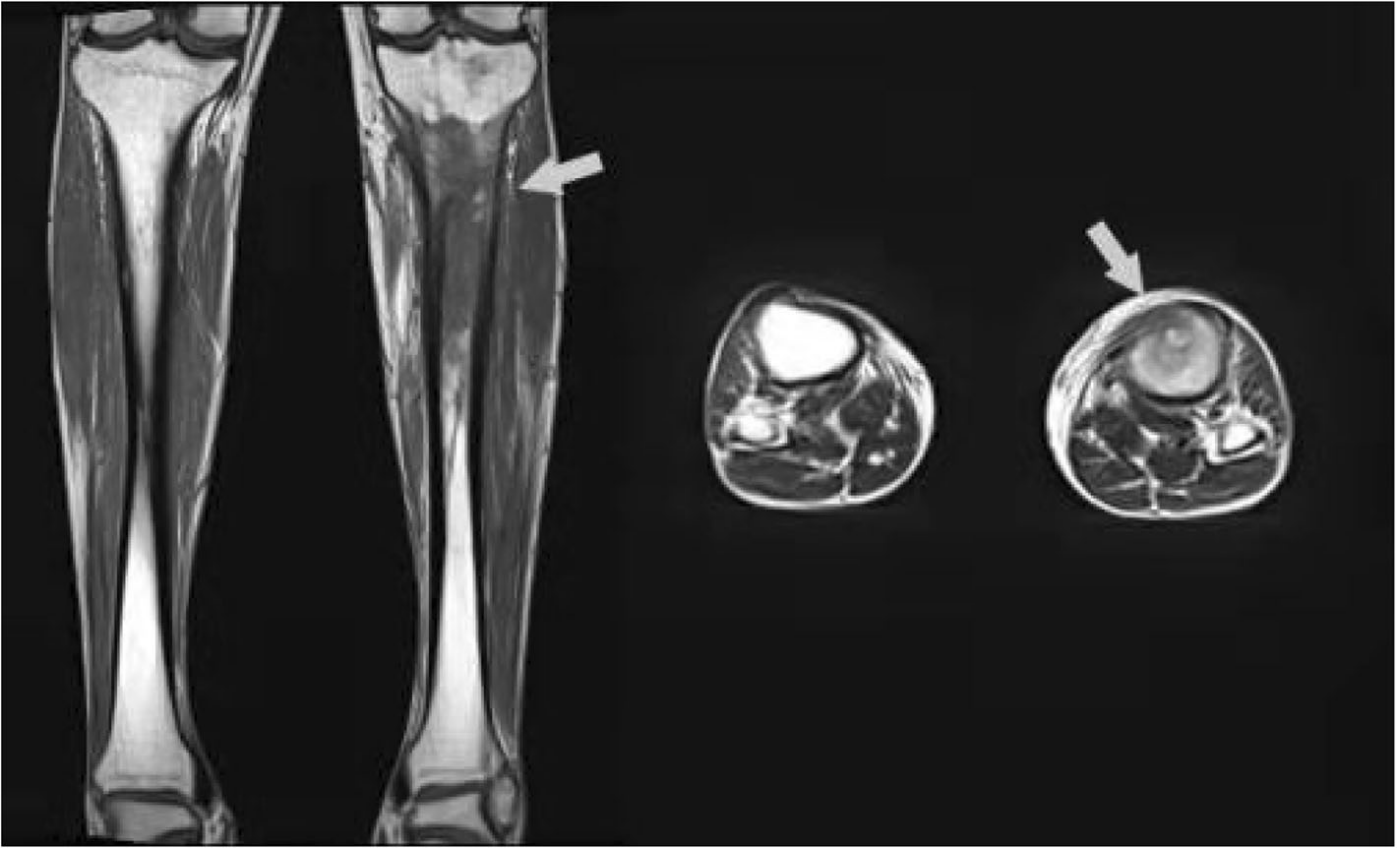
Preoperative MRI examination significantly assisted to define the diagnosis of C-M type III osteomyelitis and determine the extent of debridement

Once admitted to the inpatient department, all of patients were suggested to receive physical examinations, X-rays, and laboratory tests. For patients without implants, preoperative MRI examination was accomplished in the following days for determining the extent of infection (Fig. 1). Empirical antibiotic administration was not started until samples had been obtained for culture during surgery. Preoperative laboratory results showed, mean erythrocyte sedimentation rate (ESR) 23.22 mm/h, mean C-reactive protein (CRP) level 3.41 mg/L, and mean white blood cell (WBC) 6.71 × 10^9^/L. Intra-operative topical antibiotics of those cases included both vancomycin and gentamicin, in order to cover both Gram-positive and negative bacteria.

### Surgical technique

**Fig. 2:**
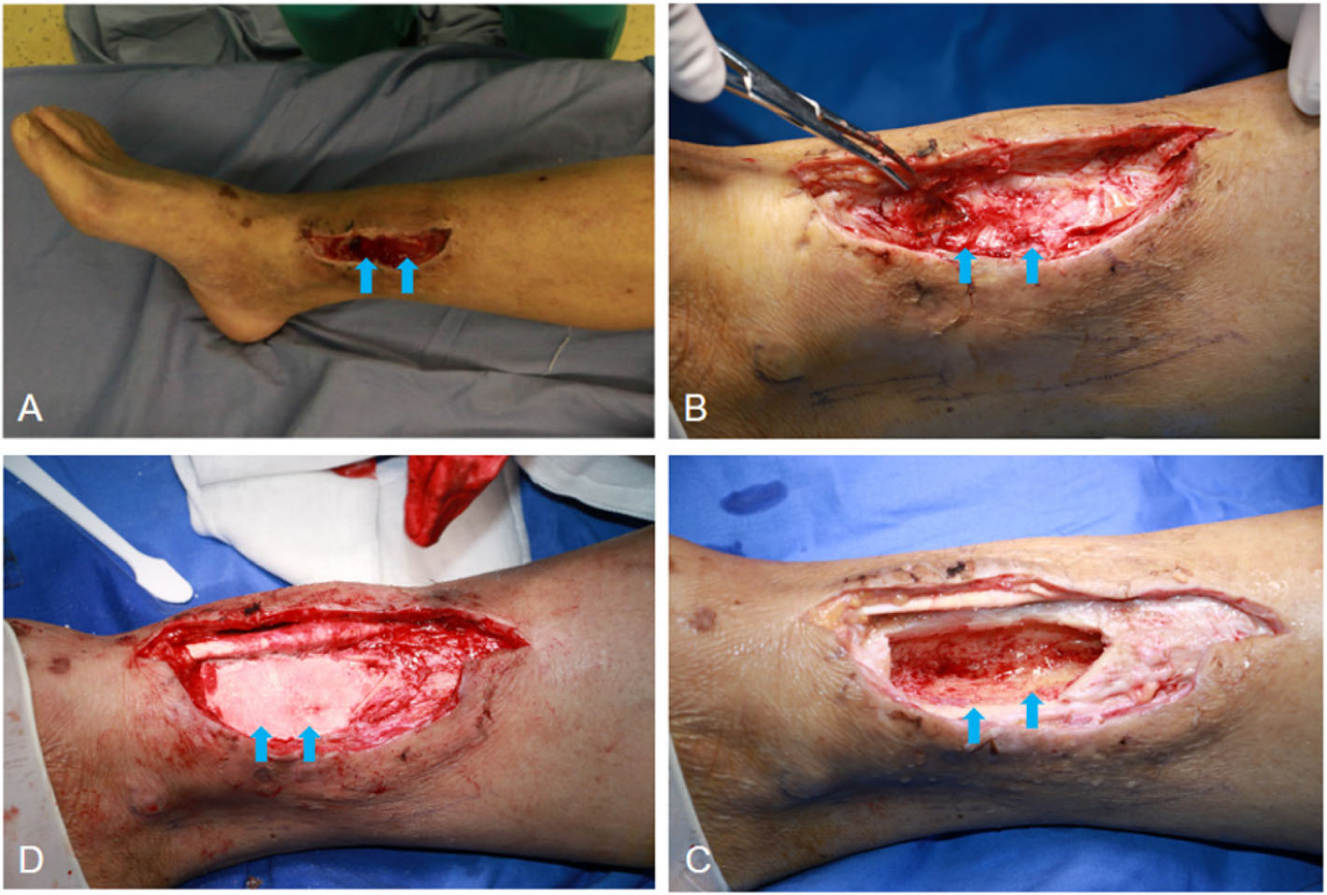
Intra-operative views of local debridement and antibiotic-loaded calcium sulfate implantation. A flap was needed to cover the soft tissue defect

Surgical procedures were carried out by experienced surgeons after spinal or nerve block anesthesia. Removal of sinus or ulcer was performed first, followed by extensive debridement of necrotic soft tissues and fibrotic scar tissues surrounding the bone area, in order to sufficiently expose the infected cortex. Any adjacent internal fixations on tibia were removed before fenestration and intramedullary debridement. Local fenestration was then carried out with the help of the high-speed burr and osteotome. The size of cortical bone fenestration depended on the extent of infection area in the bone, which was determined by the preoperative MRI examination and intra-operative presentations, such as the “Paprika sign”. Conventionally, debridement was suggested to include all infected area as well as at least 5 mm of healthy bone tissues [13], for adequately exposing the focus and preventing the infection from recurrence. Following fenestration, intramedullary debridement was started by removal of pus, inflammatory granulation, and sequestra with a rongeur. The samples were collected for bacterial culture and histological examination. After initial debridement, intramedullary reaming, irrigation, and aspiration were employed to remove the residual necrotic tissues. Eventually, a trough-like dead space in the tibia remained (Fig. 2).

Antibiotic-loaded calcium sulfate was prepared with a recommended ratio: 0.5 g vancomycin powder and 2 ml gentamicin were blended into 5 ml calcium sulfate (Stimulan, Biocomposite Ltd., UK), which were then dissolved in 0.5 ml sterile saline and embedded into the bony cavity after sufficient agitation (Fig. 2). Attributing to the limited infection of C-M III type osteomyelitis and surgical removal of involved bone, soft tissue was enough for coverage in 41 wounds; thus, those wounds were sutured directly. The other 2 wounds needed flap coverage (including 1 random flap and 1 anterolateral thigh flap) to eliminate the soft tissue losses. External fixation was applied in 13 cases with large bony defect, according to the surgeon’s experience.

### Postoperative management

After operation, broad-spectrum antibiotics were applied empirically in the first several days and exchanged to sensitive antibiotics according to results of culture for no more than 2 weeks was recommended in our study, since the high topical concentration would be attained through the degrading antibiotic-loaded calcium sulfate. Wound dressings were changed every 2 days unless excessive drainage was noted in the interval. As most of the cortical bone was preserved, full weight-bearing was encouraged once the pain of incision was eased. External fixation was removed if bone union was observed on regular X-ray examination.

### Outcomes evaluation

Normally postoperative assessment included subjective reports of patients and the objective results such as clinical examination, X-ray, and inflammatory markers. The main outcomes we focused on were infection remission, bone union, infection recurrence, and postoperative complications during the follow-up. We defined infection remission as the absence of any signs of osteomyelitis and a completely healed wound. Bone union was proved by elimination of bony cavity with the formation of new bone tissues. Osteomyelitis recurrence was defined by the presence of clinical symptoms, positive radiographic findings, and elevated inflammatory markers. In this study, we defined wound drainage without topical infection symptoms for more than 1 month as prolonged aseptic drainage.

## Results

After operation, the debridement extent of tibia was measured roughly by postoperative X-ray examination, and the mean debridement extent was about 20.9 cm^2^ (range, 2.5 to 49.8 cm^2^). The amount of antibiotic-loaded calcium sulfate used varied from patient to patient, according to the degree of bone defect and putting into consideration of patient’s economic situation as well as potential toxic effects of calcium sulfate. In our study, the mean volume of antibiotic-loaded calcium sulfate was about 28.3 cc (range, 5 to 50 cc). Instead of conventional prolonged systemic antibiotics usage (2 weeks for parental route and another 4 weeks for oral, usually), systemic antibiotics were recommended for no more than 2 weeks in our department, since the high topical concentration would be attained through the degrading antibiotic-loaded calcium sulfate. Therefore, mean systemic antibiotic usage of 42 patients was 7.0 days (range, 1 to 14 days).

Postoperative histology results of 43 limbs confirmed the diagnosis of chronic tibial osteomyelitis. Totally, 22 bacteria were isolated from 43 samples, with a positive rate of 51.2%. *Staphylococcus aureus* (50.0%, 11/22) is the most common pathogen isolated by culture, followed by *Pseudomonas aeruginosa* (13.6%, 3/22). In one case it was a polymicrobial infection and the bacterial species included *Enterococcus faecalis* and *Pseudomonas aeruginosa*, respectively. The details of the patients were presented in Table 1.

**Fig. 3:**
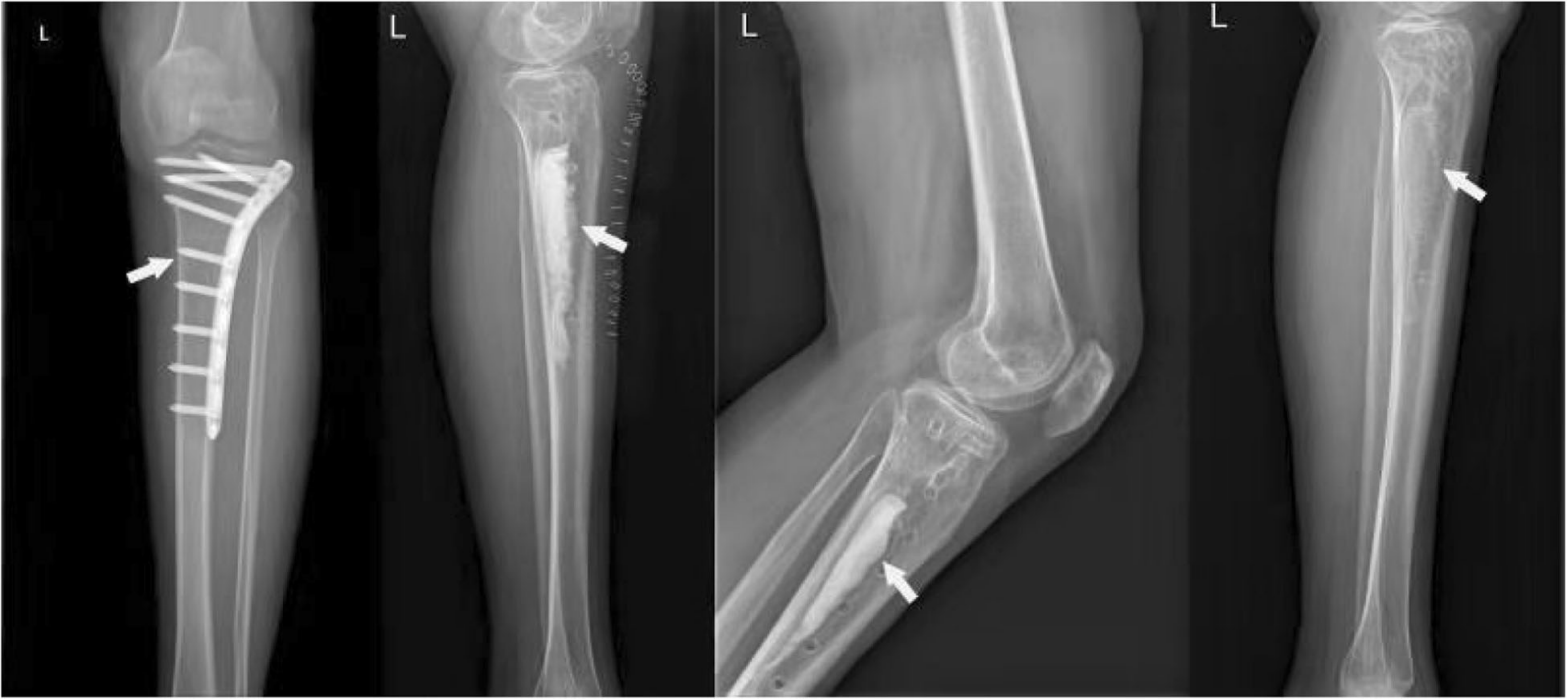
A patient with C-M type III tibial osteomyelitis received internal fixation removal and infected bone debridement, followed by implantation of antibiotic-loaded calcium sulfate. X-ray examination during the follow up showed dead space was generally substituted by new bone tissues

**Fig. 4:**
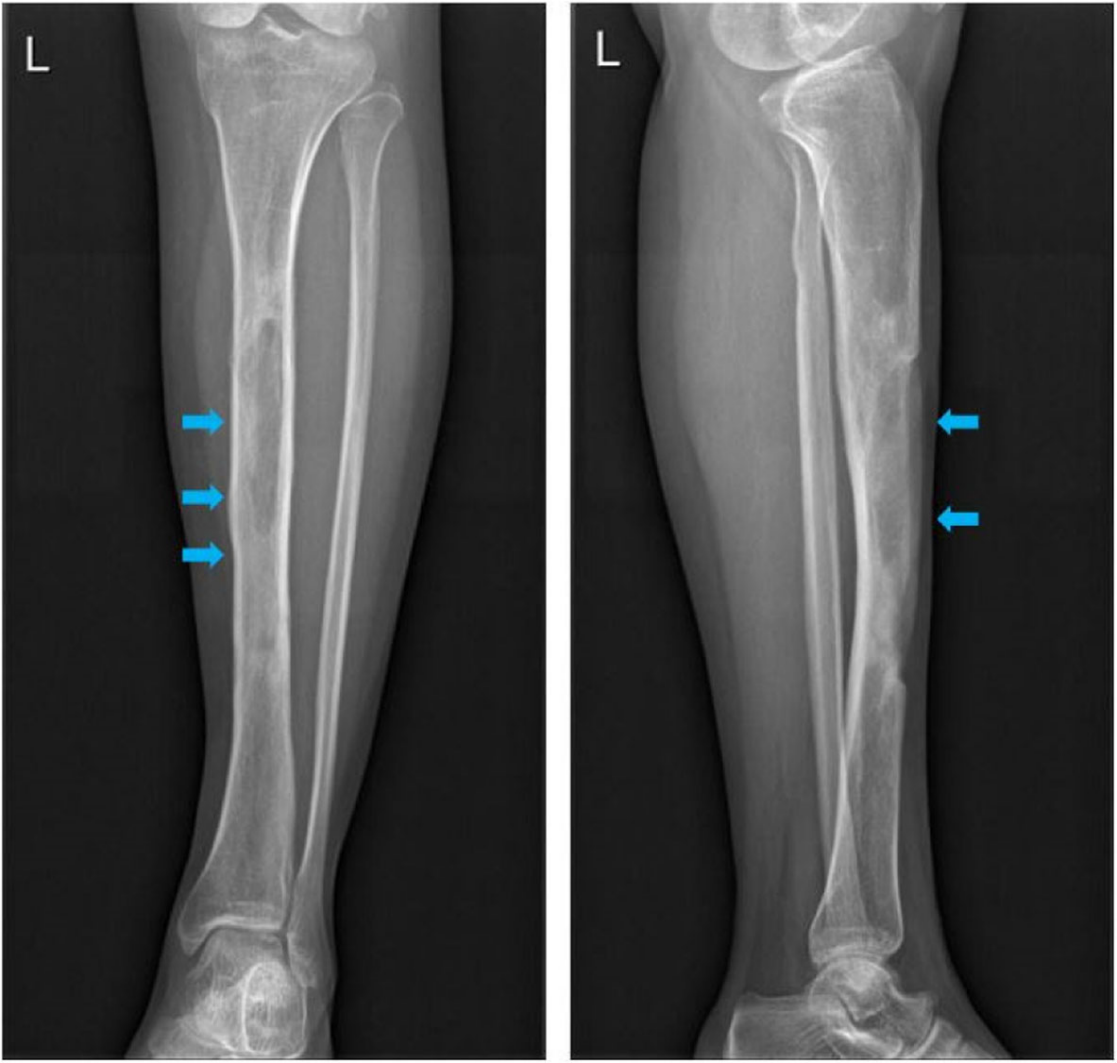
A patient received local debridement and calcium sulfate implantation. The X-ray after 6 months showed that the shape of tibia shaft was not well-restored to the initial, but no fracture was detected

During a mean follow-up of 42.8 months (12.8 to 77.5 months), we found 88.4% (38/43) limbs achieved complete infection remission without any recurrence. Only 11.6% (5/43) limbs suffered from infection recurrence within the first 3 years after operation. The management of those 5 cases was segmental bone resection and bone transport on 4 limbs and re-debridement plus flap coverage on 1 limb. Postoperative complications of those cases mainly included prolonged aseptic drainage (30%, 13/43), slight pain after a long-distance walk (10.5%, 4/38), limb weakness or discomfort (7.9%, 4/38), fibrous scar formation (5.2%,2/38), joint stiffness (2.6%, 1/38), and slight claudication (2.6%, 1/38). No case was detected with postoperative fracture or tibial bowing during the follow-up. For patients with prolonged aseptic drainage, the most acceptable and effective management was by frequently changing wound dressing, which was successfully applied to 10 limbs. Three wounds (limbs) were stubborn to the regular dressings. Therefore, surgical assistance was adopted to manage with prolonged aseptic drainage, after the infection had been well-controlled. External fixation was applied in 13 limbs with large bone loss, aiming to maintain the stability and avoid postoperative fracture. During an average fixation time of 11.8 months (3 to 29 months), no fracture or tibial bowing effect was detected. According to the latest X-ray examination, a generally satisfying bone formation was achieved in all healed limbs (Fig. 3), although the shape of the tibia was not restored to their initial form in 17 cases (Fig. 4).

## Discussion

C-M Type III chronic tibial osteomyelitis as a localized infection involves full-thickness cortical bone as well as the medullary tissue, which may develop to a diffused infection if not well-managed. However, impaired local vascular condition on bone sclerosis and sequestrum makes it difficult for parental antimicrobial therapy alone to achieve satisfying local effects, even with a prolonged course of application. Besides, *Staphylococcus aureus* as the most frequently detected pathogen in osteomyelitis is prone to produce biofilm, which irreversibly binds to the surface of the bone and on the internal implants, causing the infection to be stubborn and hard to eliminate [14]. To address this embarrassing situation, surgical intervention is the cornerstone for the treatment of osteomyelitis; it not only removes the necrotic tissues but destroys the biofilms caused by pathogens, therefore stimulating the local blood supply and enhancing the antimicrobial effects of antibiotics.

For localized tibial osteomyelitis, surgical principles may be interpreted as the combination of “radicalization” and “limitation”. The principle of “radicalization” requires thorough removal of necrotic tissues and some adjacent healthy bone, to create a relatively clean wound for following reconstructive steps, while the principle of “limitation” requires to preserve as much healthy bone as possible (under the premise of thorough debridement, of course), to prevent the complication of postoperative fractures or deformities. Therefore, conventional aggressive debridement technique for diffused tibial osteomyelitis, segmental bone resection, is unsuitable for the treatment of localized tibial osteomyelitis. To match the demands mentioned above, local debridement (deroofing associated with the intramedullary debridement) is introduced as the treatment of localized tibial osteomyelitis, and the effects are satisfying. In a former study, Beals and Bryant reported 30 consecutive cases with tibial osteomyelitis, which included 1 case with Cierny-Mader type IIIA and the other 8 cases with type IIIB. The treatment methods of localized osteomyelitis included local debridement only in 5 cases, local debridement and muscle flaps coverage in 2 cases, and multiple debridement and posterior-lateral bone graft in 1 case. All of the cases achieved good outcomes at the end of follow-up [15]. Kinik and Karaduman treated 26 cases with chronic localized osteomyelitis in his work. Those patients were treated with deroofing and local debridement, irrigation, vancomycin-impregnated polymethylmethacrylate (PMMA) beads implantation at first stage, followed by re-debridement, and PMMA beads removal 6 to 8 weeks later. Within a mean follow-up of 3.6 years, all patients achieved infection remission with normal clinical parameters, even though 3 patients had to receive re-debridement in the interval [6].

In our study, this classical local debridement technique was also employed, for effective removal of necrotic tissues. The main difference is the replacement of temporary antibiotic-impregnated PMMA beads or autologous tissues grafts by biodegradable antibiotic-impregnated calcium sulfate, which undoubtedly has its unique advantages. Primarily, the predictable high local antibiotic concentration (hundreds to thousands times higher than minimum inhibitory concentration (MIC) in first 24 to 48 h) and comparatively long therapeutic duration (several weeks to months) [5, 16, 17] provided by the degrading antibiotic-loaded calcium sulfate undoubtedly eliminates more residual pathogens while significantly shortens the conventional duration of systemic antibiotics administration. Moreover, similar to PMMA, the well-reported osteo-conductivity of calcium sulfate provides a crystalline structure for the osteoblasts perivascular mesenchymal tissues and osteoprogenitors, along which osteoblasts and the others crawl easily and eventually achieve the self-repair without autogenous bone grafts [10, 18]. While combined with the biodegradation characteristic of calcium sulfate, it allows orthopedists to accomplish debridement and reconstruction within one operation only, significantly avoiding the redundant reconstructive procedures.

With application of antibiotic-loaded calcium sulfate implantation, generally satisfying outcomes were achieved in our study at the end of follow-up. This was well-illustrated by the fact that 88.4% of our patients achieved infection remission after first operation. Even for patients with recurrence, a chance for re-debridement could be preserved and managed accordingly. This extremely high remission rates were similar to the previous study by Ferguson et al., who managed 144 cases with type III chronic osteomyelitis (195 cases, totally) using local debridement and implantation of tobramycin-contained calcium sulfate beads. Their records showed only 11 cases (7.6%) of type III chronic osteomyelitis recurred within a mean follow-up of 3.7 years, while most of the cases were successfully managed by re-debridement and antibiotics usage [19]. However, although the samples of their study were relatively large, it contained a variety of infection sites (femur, tibia, humerus, radius, ulnar, pelvic, and even calcaneus) and the four stage types of Cierny-Mader classification. Thus, their study ineluctably lacked an in-depth discussion on a single type and site of chronic osteomyelitis. In another comparative study, Ferrando et al. received a similar result after using topical antiobiotic delivery system on chronic osteomyelits. They compared the efficacy of bioglass (BAG-S53P4) and calcium sulfate antibiotic beads in the treatment of chronic osteomyelitis. For 13 patients (7 on tibia) with antibiotic-loaded calcium sulfate implantation, 12 patients achieved infection remission during the follow-up [20].

While infection elimination was effective, associated complications were also of concern. Prolonged aseptic drainage was the most frequent recorded complication in our study, with a relatively high rate of 30.0%. This incidence was variant from person to person, primarily depending on the volume of implanted calcium sulfate and the abundance of soft tissues coverage. To our study, poor soft tissue coverage in the medial surface of tibia, scar formation surrounding to the focus, and large volume implantation of calcium sulfate might interpret the high incidence of postoperative drainage. Kallala et al. previously concluded a 4.2% incidence of prolonged aseptic drainage after calcium sulfate implantation [8], compared to the higher incidence of 15.4% [19], 33% [21], and 27% [22] in other studies. If not accompanied with typical presentations, positive inflammatory markers, and imaging examination, prolonged aseptic drainage alone should not be considered as a sign of infection recurrence, since it is a common presentation produced by the gradual degradation of calcium sulfate. Though the liquid is sterile, however, immediate management is of great necessity, or a soggy gauze is prone to cause wound infection. Generally, it can be well-managed with regular dressing and wound care. Other effective methods to prevent from aseptic drainage might include employing a flap or muscle flap when the soft tissue of tibia is too poor to cover, using lateral incision instead of medial incision, and implanting less antibiotic-loaded calcium sulfate if a medial incision is applied. Another complication-needing attention is the, not very satisfying, self-restored shape of tibia in 17 cases during the follow-up, which was well-illustrated on X-rays. We suspect this situation might be attributed to the unconfirmed relation between degradation of calcium sulfate and growth of osteoblasts, yet there is no evidence in former studies to support our hypothesis. Although the shape of the tibia in these cases was not well-restored, however, there was no operation-related fracture or tibial bowing effect was recorded during the follow-up, which means that the tibia still has enough strength to bear the weight.

To our best knowledge, separate study for evaluating C-M type III tibial osteomyelitis is still rare. Our study might be the first to assess the outcomes of this technique for a single-stage treatment of chronic localized tibial osteomyelitis, with a larger number of patients. The drawbacks of our study were also obvious. To begin with, its retrospective characteristic means only limited information was available. Thus, it inevitably reduces the credibility of our study. In addition, outcomes of this study were not compared with those of other surgical methods, a comparative study is necessary to be carried out. Finally, we included osteomyelitis caused by fracture and related treatment, hematogenous transmission, and penetration infection in our study. However, no matter whether chronic localized osteomyelitis was caused by fracture, hematogenous transmission, or continuous penetration, the treatment protocols for localized osteomyelitis were similar—local debridement plus antibiotics-loaded calcium sulfate implantation. So, we think that including all these types in this study was rational.

## Conclusion

Local debridement combined with antibiotic-loaded calcium sulfate as a single-stage treatment is effective in treating chronic localized tibial osteomyelitis.

## Data Availability

The datasets used and analyzed during the current study are available from the corresponding author on reasonable request.

## Abbreviations

C-M: Cierny-Mader
PMMA: Polymethylmethacrylate
MIC: Minimum inhibitory concentration
VAC: Vacuum-assisted closure
ESR: Erythrocyte sedimentation rate
CRP: C-reactive protein
WBC: White blood cell
MRI: Magnetic resonance imaging

## References

[1] Jiang N, Ma Y, Jiang Y, Zhao X, Xie G, Hu Y, Qin C, Yu B (2015). Clinical characteristics and treatment of extremity chronic osteomyelitis in Southern China. Medicine.

[2] Bhattacharya R, Kundu B, Nandi SK, Basu D (2013). Systematic approach to treat chronic osteomyelitis through localized drug delivery system: bench to bed side. Mater Sci Eng C.

[3] Cierny GR, Mader JT, Penninck JJ (2003). A clinical staging system for adult osteomyelitis. Clin Orthop Relat Res.

[4] Parsons B, Strauss E (2004). Surgical management of chronic osteomyelitis. Am J Surg.

[5] Wahl P, Guidi M, Benninger E, Ronn K, Gautier E, Buclin T, Magnin JL, Livio F (2017). The levels of vancomycin in the blood and the wound after the local treatment of bone and soft-tissue infection with antibiotic-loaded calcium sulphate as carrier material. Bone Joint J.

[6] Kinik H, Karaduman M (2008). Cierny-Mader type III chronic osteomyelitis: the results of patients treated with debridement, irrigation, vancomycin beads and systemic antibiotics. Int Orthop.

[7] Zumiotti AV, Teng HW, Ferreira MC (2003). Treatment of post-traumatic tibial osteomyelitis using microsurgical flaps. J Reconstr Microsurg.

[8] Kallala R, Harris WE, Ibrahim M, Dipane M, McPherson E (2018). Use of stimulan absorbable calcium sulphate beads in revision lower limb arthroplasty: safety profile and complication rates. Bone Joint Res.

[9] Qin CH, Zhang HA, Chee YH, Pitarini A, Adem AA (2019). Comparison of the use of antibiotic-loaded calcium sulphate and wound irrigation-suction in the treatment of lower limb chronic osteomyelitis. Injury.

[10] Beuerlein MJ, McKee MD (2010). Calcium sulfates: what is the evidence?. J Orthop Trauma.

[11] De Long WJ, Einhorn TA, Koval K, McKee M, Smith W, Sanders R, Watson T (2007). Bone grafts and bone graft substitutes in orthopaedic trauma surgery. A critical analysis. J Bone Joint Surg Am.

[12] Yikemu X, Tuxun A, Nuermaimaiti M, Abudukeyimu A, Shayiti A (2019). Effects of vacuum sealing drainage combined with Ilizarov bone transport technique in the treatment of tibial traumatic osteomyelitis. Med Sci Monitor.

[13] Simpson AH, Deakin M, Latham JM (2001). Chronic osteomyelitis. The effect of the extent of surgical resection on infection-free survival. J Bone Joint Surg Br.

[14] Donlan RM (2002). Biofilms: microbial life on surfaces. Emerg Infect Dis.

[15] Beals RK, Bryant RE: The treatment of chronic open osteomyelitis of the tibia in adults. Clin Orthop Relat R 2005, &NA;(433):212-217. 10.1097/01.blo.0000150462.41498.fe 15805960

[16] Miclau T, Dahners LE, Lindsey RW (1993). In vitro pharmacokinetics of antibiotic release from locally implantable materials. J Orthop Res.

[17] Cooper JJ, Florance H, McKinnon JL, Laycock PA, Aiken SS (2016). Elution profiles of tobramycin and vancomycin from high-purity calcium sulphate beads incubated in a range of simulated body fluids. J Biomater Appl.

[18] Turner TM, Urban RM, Hall DJ, Chye PC, Segreti J, Gitelis S (2005). Local and systemic levels of tobramycin delivered from calcium sulfate bone graft substitute pellets. Clin Orthop Relat Res.

[19] Ferguson JY, Dudareva M, Riley ND, Stubbs D, Atkins BL, McNally MA (2014). The use of a biodegradable antibiotic-loaded calcium sulphate carrier containing tobramycin for the treatment of chronic osteomyelitis: a series of 195 cases. Bone Joint J.

[20] Ferrando A, Part J, Baeza J (2017). Treatment of cavitary bone defects in chronic osteomyelitis: bioactive glass S53P4 vs. calcium sulphate antibiotic beads. Journal Of Bone And Joint Infection.

[21] Badie AA, Arafa MS (2019). One-stage surgery for adult chronic osteomyelitis: concomitant use of antibiotic-loaded calcium sulphate and bone marrow aspirate. Int Orthop.

[22] Romano CL, Logoluso N, Meani E, Romano D, De Vecchi E, Vassena C, Drago L (2014). A comparative study of the use of bioactive glass S53P4 and antibiotic-loaded calcium-based bone substitutes in the treatment of chronic osteomyelitis: a retrospective comparative study. Bone Joint J.

